# Comparison of the CTDI and AAPM report No. 111 methodology in adult, adolescent, and child head phantoms for MSCT and dental CBCT scanners

**DOI:** 10.1117/1.JMI.4.3.031212

**Published:** 2017-09-29

**Authors:** Celina L. Li, Yogesh Thakur, Nancy L. Ford

**Affiliations:** a University of British Columbia, Faculty of Science, Department of Physics and Astronomy, Vancouver, British Columbia, Canada; b Lower Mainland Medical Imaging Services, Vancouver Coastal Health Authority, Vancouver, British Columbia, Canada; c University of British Columbia, Faculty of Medicine, Department of Radiology, Vancouver, British Columbia, Canada; d University of British Columbia, Faculty of Dentistry, Department of Oral Biological and Medical Sciences, Vancouver, British Columbia, Canada

**Keywords:** dosimetry, cone beam computed tomography, computed tomography dose index, pediatric head phantom, equilibrium dose

## Abstract

This study investigates the dosimetry methodology proposed by the American Association of Physicists in Medicine (AAPM) task group 111 and compares with the computed tomography dose index (CTDI) method and the SEDENTEXCT DI method on one clinical multislice CT and two dental cone beam CT (CBCT) scanners using adult, adolescent, and child head phantoms. Following the AAPM method, the normalized (100 mAs) equilibrium doses ($D_{\mathrm{eq}}$) for Toshiba Aquilion One MSCT computed using dose measurements from the central hole of the phantom ($D_{\mathrm{eq},c}$), the peripheral hole of the phantom, ($D_{\mathrm{eq},p}$), and by the ${\mathrm{CTDI}}_{w}$ equation ($D_{\mathrm{eq},w}$) are in the range from 20 to 25 mGy. For i-CAT Next Generation dental CBCT, the normalized $D_{\mathrm{eq},c}$, $D_{\mathrm{eq},p}$, $D_{\mathrm{eq},w}$, and $D_{\mathrm{eq}}^{\prime }s$ by the two SEDENTEXCT DI methods are in the range from 12 to 15 mGy. Fitting the AAPM equation is not possible for the limited scan lengths available on the CS 9300 dental CBCT. This study offers a simple CTDI-like measurement that can approximate the AAPM $D_{\mathrm{eq}}$ in clinical CBCT scanners capable of providing four or more scan lengths.

## 1 Introduction

The computed tomography dose index (CTDI) measurement is the standard dosimetry method that uses a 10-cm pencil ionization chamber to approximate dose output for a single axial scan.^1^^–^^9^ The weighted CTDI (${\mathrm{CTDI}}_{w}$) combines CTDI measurements at the phantom center and periphery (${\mathrm{CTDI}}_{c}$ and ${\mathrm{CTDI}}_{p}$, respectively)^1^ to generate a weighted dose index. Phantom sizes are typically 32 or 16 cm in diameter, representing the nominal effective diameter of adult body and head sizes.^4^ With the advent of advanced computed tomography (CT), including multislice CT (MSCT) and cone beam CT (CBCT), which have longer scan lengths, the CTDI dosimetry method for CT dose assessment becomes unreliable because it underestimates scatter radiation beyond the length of the 10-cm pencil ionization chamber and therefore underestimates the cumulative dose at the phantom central plane (z=0).^2^^–^^9^

Furthermore, with the CBCT technology emerging in private dental practice, stringent dose assessments are required because dental CBCT scanners deliver considerably higher radiation doses as compared to other conventional two-dimensional dental radiographic machines.^10^^,^^11^ This is very concerning for patients and, particularly, pediatric patients in dental practice, since a sophisticated guideline for radiation management with dental CBCT scanners is not yet widely adopted and private dental care operates independently from hospitals, with limited to no support from medical physicists for quality assurance.^10^^,^^11^ The SEDENTEXCT (safety and efficacy of a new and emerging dental x-ray modality) guidelines have illustrated principles for use of CBCT in dentistry, including justification and optimization of x-ray exposures.^12^

To overcome difficulties posed by new CT technologies and correct dose underestimation produced by the CTDI method,^4^ the American Association of Physicists in Medicine (AAPM) task group 111 proposed a new measurement paradigm for CBCT acquisition in 2010. Based on the new AAPM methodology, Deman et al.^2^ examined its application over multiple x-ray modalities, and extended the method to approximate doses at off-centered planes (z≠0). In this study, we utilize the same methods to characterize dose profiles in adult, adolescent, and child head phantoms and compare with the CTDI method and dental dose index method to provide insights for developing a robust dose assessment in MSCT and dental CBCT scanners.

## 2 Materials and Methods

### 2.1 Computed Tomography Imaging Scanners, Radiation Dosimeters, and Phantoms

**Fig. 1:**
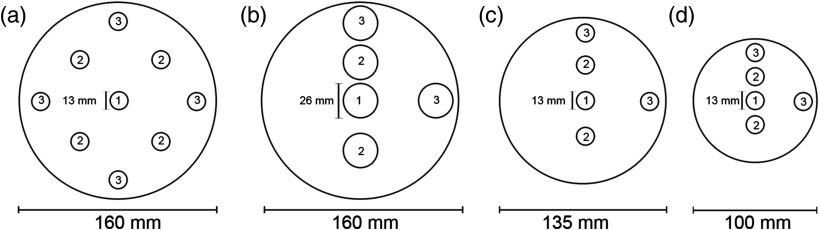
Phantom schematics from the transverse plane. Center positions are labeled with number 1, midhole positions are labeled with number 2, and peripheral positions are labeled with number 3. (a) The Food and Drug Administration (FDA) CTDI adult head PMMA phantom, (b) the SEDENTEXCT DI dose index phantom, (c) the custom-built adolescent head PMMA phantom, and (d) the custom-built child head PMMA phantom.

**Fig. 2:**
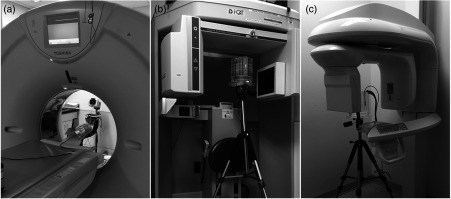
Experimental setups for all three CT imaging scanners. (a) The adolescent head phantom positioned within the head holder of the Toshiba Aquilion One scanner with the thimble chamber inserted into its posterior hole; the towel is used to immobilize the phantom. (b) The experimental setup of the SEDENTEXCT DI dose index phantom positioned on the tripod in the i-CAT Next Generation CBCT scanner with the thimble chamber inserted into its anterior hole and aligned to z=−80  mm. (c) The experimental setup of the SEDENTEXCT DI dose index phantom positioned on the tripod in the CS 9300 CBCT scanner with the thimble chamber inserted into its anterior hole and aligned to z=+40  mm.

This study has examined three CT imaging scanners, including one common clinical MSCT scanner (Toshiba Aquilion^™^ One, Toshiba America Medical Systems, Inc., Tustin, California) and two dental CBCT scanners (i-CAT Next Generation CBCT, Imaging Sciences International, LLC, Hatfield, Pennsylvania and CS 9300 CBCT, Carestream Dental LLC, Atlanta, Georgia). Using the CBCT acquisition, doses were measured in adult, adolescent, and child head phantoms for each CT imaging scanner. Figures 1(a) and 1(b) show schematics of two adult head phantoms that were included to represent the average adult head, which are the FDA CTDI phantom (160-mm diameter and 150-mm length) manufactured by Computerized Imaging Reference System, Inc. (Norfolk, Virginia) for use with the MSCT scanner and the SEDENTEXCT DI dose index phantom (160-mm diameter and 162-mm length) manufactured by Leeds Test Objects Ltd. (Boroughbridge, North Yorkshire) for use with the dental CBCT scanners. Since the SEDENTEXCT DI dose index phantom has 26-mm diameter holes, we used a customized sheath to fill the outer part of the hole with polymethyl methacrylate (PMMA), which fits more closely to the ion chamber. Two head phantoms were designed in our lab and custom-built (British Columbia Cancer Agency, Genome Sciences Centre, Vancouver, BC, Canada) to simulate pediatric patients [Figs. 1(c) and 1(d)]. The adolescent head phantom, which measures 135 mm in diameter and 150 mm in length, aims to represent a 12-year-old patient, corresponding to the age at entry into orthodontic treatment.^13^ The child head phantom, which measures 100 mm in diameter and 150 mm in length, represents a 5-year-old patient, corresponding to the youngest age for receiving dental CBCT scans in the local children’s hospital dental department.^13^ All phantoms are made of PMMA, which has a density of $1.20\pm 0.01\;g\;{\mathrm{cm}}^{-3}$ similar to that of human soft tissue. Figure 2 shows an example of the actual experimental setups for all three CT imaging scanners.

As proposed by the AAPM report No. 111,^4^ a calibrated $0.6\;{\mathrm{cm}}^{3}$ thimble ionization chamber (active length: 19.7±1  mm; 10x6-0.6CT, Radcal Corporation, Monrovia, California) along with a Radcal radiation dosimeter (AccuDose, Radcal Corporation, Monrovia, California) was used to measure the radiation dose in all phantoms;^4^ the same thimble chamber setup was also used for the SEDENTEXCT DI measurements. For the CTDI measurements, a 10-cm pencil ionization chamber (Unfors Raysafe AB, Sweden) was used.

### 2.2 Dose Estimation by the CTDI, the AAPM Report 111, and the SEDENTEXCT DI Methods

For the standard CTDI method, the 10-cm pencil chamber was utilized to measure doses in the central (${\mathrm{CTDI}}_{c}$) and the peripheral (${\mathrm{CTDI}}_{p}$) holes of the phantom at the central plane (z=0), and ${\mathrm{CTDI}}_{w}$ [Eq. (1)] was computed for all CT imaging scanners.

$${\mathrm{CTDI}}_{W}=\frac{1}{3}{\mathrm{CTDI}}_{C}+\frac{2}{3}{\mathrm{CTDI}}_{P}.$$ (1)

Doses measured at z=0 from all phantoms by the AAPM method were fitted using the statistical programming language R (The R Foundationhttps://www.r-project.org/, Vienna, Austria) with Eq. (2) proposed by Dixon et al.^4^

$$D_{L}(z=0)\approx D_{\mathrm{eq}}[(1-\alpha )+\alpha (1-e^{-4\frac{L}{L_{\mathrm{eq}}}})]=D_{\mathrm{eq}}(1-\alpha e^{-4\frac{L}{L_{\mathrm{eq}}}}),$$ (2)

$$D_{L}{\left(z=0\right)}_{\text{primary}}=D_{\mathrm{eq}}(1-\alpha ),$$ (3)

$$D_{L}{\left(z=0\right)}_{\text{scatter}}=D_{\mathrm{eq}}\alpha (1-e^{-4\frac{L}{L_{\mathrm{eq}}}}),$$ (4)

where L is the scan length, and $D_{L}$ (z=0) represents the measured dose over the entire scan length L centered at z=0. $D_{\mathrm{eq}}$, $L_{\mathrm{eq}}$, and α represent the equilibrium dose, the equilibrium length at which the measured dose becomes asymptotic to the equilibrium dose value, and the radiation factor that distinguishes between the contribution of primary radiation [Eq. (3)] and scatter radiation [Eq. (4)], respectively. The values of these three parameters were computed by the fit. The peripheral measurements in the Toshiba MSCT scanner were obtained by averaging the anterior and posterior measurements. Measurements in the right and left hole were not included in the average dose calculation, since we operated the MSCT to perform a complete rotation that produced similar dose values at the right and the left due to the shape of the CT head holder. The peripheral measurements in the dental scanners were computed by averaging the dose measurements taken in the anterior, posterior, right, and left holes of the phantom because the dental CBCT scanners may perform scans using a partial rotation; the partial rotation is used for certain fields of view with no option to complete a full rotation, in contrast to the MSCT, where the partial scan mode can be used as desired. The central and the peripheral dose measurements are fitted individually with Eq. (2) to obtain the central $D_{\mathrm{eq}}$ ($D_{\mathrm{eq},c}$) and the peripheral $D_{\mathrm{eq}}$ ($D_{\mathrm{eq},p}$). Furthermore, the ${\mathrm{CTDI}}_{w}$ equation was utilized to calculate a weighted dose index using dose measurements by the thimble chamber from the central and the peripheral holes of the phantom for each scan length, and several such indices corresponding to their respective scan lengths were also fitted with Eq. (2) to obtain an equilibrium dose value, namely the “weighted $D_{\mathrm{eq}}$ ($D_{\mathrm{eq},w}$),” for comparison with $D_{\mathrm{eq},c}$ and $D_{\mathrm{eq},p}$. In addition, the dose profiles along the z-axis were estimated and plotted using the three parameters $D_{\mathrm{eq}}$, $L_{\mathrm{eq}}$, and α obtained from the AAPM fit described above, and the equations are shown^2^

$$\text{For}\;-\infty <z<-\frac{L}{2},\;D(z=l)=\frac{1}{2}\alpha D_{\mathrm{eq}}[1-e^{\frac{-4(L-2l)}{L_{\mathrm{eq}}}}]-\frac{1}{2}\alpha D_{\mathrm{eq}}[1-e^{\frac{4(L+2l)}{L_{\mathrm{eq}}}}];\;\text{For}\;-\frac{L}{2}<z<\frac{L}{2},\;D(z=l)=\frac{1}{2}\alpha D_{\mathrm{eq}}[1-e^{\frac{-4(L-2l)}{L_{\mathrm{eq}}}}]+\frac{1}{2}\alpha D_{\mathrm{eq}}[1-e^{\frac{-4(L+2l)}{L_{\mathrm{eq}}}}]+D_{\mathrm{eq}}(1-\alpha );\;\text{For}\;\frac{L}{2}<z<\infty ,\;D(z=l)=-\frac{1}{2}\alpha D_{\mathrm{eq}}[1-e^{\frac{-4(2l-L)}{L_{\mathrm{eq}}}}]+\frac{1}{2}\alpha D_{\mathrm{eq}}[1-e^{\frac{-4(L+2l)}{L_{\mathrm{eq}}}}].$$ (5)

The SEDENTEXCT DI method introduces two types of dose index calculations for dental CT imaging scanners, which are calculating dose measurements along the diameter of the phantom (dose index 1) and dose measurements along the periphery of the phantom (dose index 2).^14^ For dose measurements along the diameter, the measuring diameter is determined by the gradient of dose distribution. The average of dose measurements along the diameter is calculated to represent the diameter dose index (Sedentex-DI1). The second dose index (Sedentex-DI2) uses dose measurements from the central and the peripheral positions as shown in

$${\mathrm{DI}}_{\text{periphery}}=\frac{D_{c}+D_{p}}{2},$$ (6)

where $D_{c}$ is the dose measured in the central hole of the phantom and $D_{p}$ is the average of all peripheral dose measurements.^14^ The SEDENTEXTCT DI method is used only with the dental scanners to calculate Sedentex-DI1’s and Sedentex-DI2’s for all scan lengths, which are then fitted with the AAPM equation to obtain Sedentex-DI1 $D_{\mathrm{eq}}$ and Sedentex-DI2 $D_{\mathrm{eq}}$, respectively.

### 2.3 Dose Measurements in the Multislice Computed Tomography Scanner

For the MSCT scanner, doses were measured using the FDA CTDI phantom to represent an average adult head and the two custom-built phantoms to represent an adolescent and a child head; the SEDENTEXCT DI dose index phantom was not used in the MSCT scanner. Each phantom was placed in the scanner to imitate patient positioning during a head CT scan. In particular, to measure doses at z=0, the 2-cm thimble chamber was inserted into the central hole and aligned to z=0 of the phantom. The phantom central axis was then aligned to the isocenter of the scanner to obtain the central dose measurements. The probe was then moved to the anterior and the posterior holes to obtain the peripheral dose measurements. For the adolescent and child phantoms, the phantoms were physically rotated to position the peripheral hole in the anterior, posterior, left, and right locations since the phantoms do not have the complete set of holes. For measuring the ${\mathrm{CTDI}}_{100}$ values, the 10-cm pencil chamber was positioned with the same setup as with the 2-cm thimble chamber in which the dose measurements taken from the central and the peripheral holes are the ${\mathrm{CTDI}}_{c}$ and ${\mathrm{CTDI}}_{p}$, respectively. The scan lengths (L) used included 4, 32, 40, 60, 80, 100, 120, 140, and 160 mm for the adult phantom. Since the dose reached an asymptote past 100 mm, we only performed measurements for scan lengths of 4, 32, 40, 60, 80, and 100 mm for the adolescent and child phantoms. The acquisition parameters were 120 kV, 300 mA, and 1-s exposure time.

**Fig. 3:**
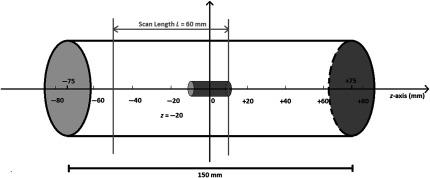
Experimental setup of the phantom (schematic). In the example, the ionization chamber is centered inside the phantom, and the center of L is aligned to z=−20  mm with a scan length of 60 mm.

Dose measurements at off-centered planes (z≠0) were performed with the same experimental setup, scan lengths, and parameters. The midpoint of each L was aligned with various locations (z=0, ±20, ±40, ±60, ±80  mm) along the phantom z-axis with the thimble chamber fixed at z=0 inside the phantom to measure doses at off-centered planes. The hole positions included the center and the anterior positions as instructed by the AAPM report No. 111.^2^^,^^4^ Starting from one extreme, an increment of 20 mm was adopted to capture scatter dose and obtain a full dose profile along the z-axis. Figure 3 illustrates the schematic diagram explaining the setup with L=60  mm centered at z=−20  mm.

### 2.4 Dose Measurements in the Dental Cone Beam Computed Tomography Scanners

For the two dental CBCT scanners, doses were measured using the SEDENTEXCT DI dose index phantom to represent an average adult head and the two custom-built phantoms to represent an adolescent and a child head; the FDA CTDI phantom was not used in the dental CBCT scanners. Each phantom was positioned onto a height-adjustable tripod and aligned within the field of view (FOV) using the patient positioning lasers. The geometrical orientation of the dental CBCT scanners has a vertical rotational axis (z-axis), with the positive direction toward the crown and negative direction toward the feet. For i-CAT Next Generation (i-CAT NG) dental CBCT scanner, the scan lengths (L) used are 40, 60, 80, 100, 110, and 130 mm, with 16-cm axial coverage. The acquisition parameters include 120 kVp and 18.54 mAs. For the CS 9300 dental CBCT scanner, the scan lengths (L) used include 60, 110, and 135 mm, with 17-cm axial coverage. The acquisition parameters are 80 kVp and 25.2 mAs for child, 85 kVp and 25.6 mAs for adolescent, and 90 kVp and 45.2 mAs for adult, respectively.

For both dental CBCT scanners, doses were measured at both the central plane (z=0) and the off-centered planes (z≠0) for all phantoms. For all three phantoms, we rotated the phantom to obtain measurements in the desired locations, as the full set of holes was not available. The 2-cm thimble chamber was centered within the phantom height in the central hole and in the four peripheral holes of the phantom to obtain central and peripheral dose measurements at z=0, respectively. As with the MSCT scanner, the off-axis doses measured for the two dental CBCT scanners were also obtained by aligning the midpoint of each L to z=0, ±20, ±40, ±60, and ±80  mm, respectively. The SEDENTEXCT DI measurements are obtained by measuring doses using the thimble chamber in the five holes along the gradient diameter for DI1 calculation, and in the center and peripheral holes for DI2 calculation.

## 3 Results

### 3.1 Multislice Computed Tomography Scanner

**Fig. 4:**
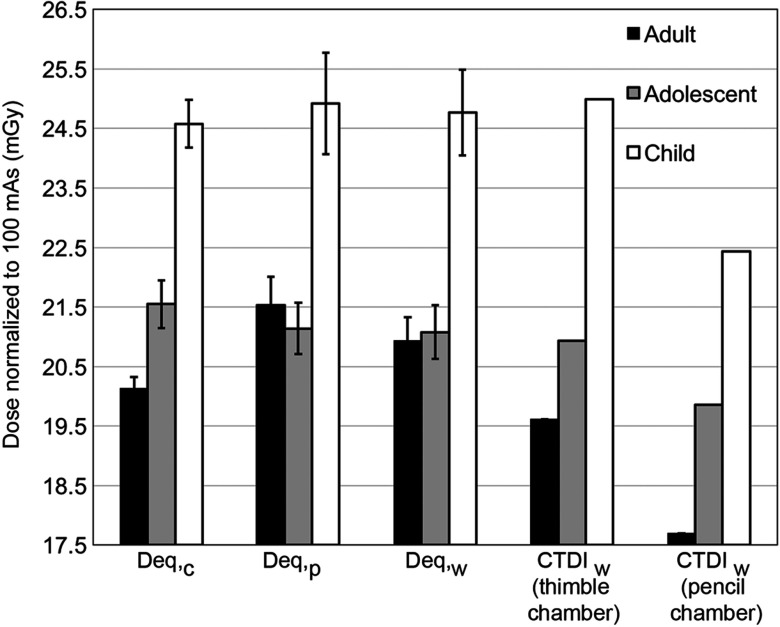
Summary of all the results obtained using the AAPM method on the Toshiba MSCT. $D_{\mathrm{eq},c}$, $D_{\mathrm{eq},p}$, and $D_{\mathrm{eq},w}$ (actual values are summarized in Table 2) are calculated from the AAPM fit [Eq. (2)] with error bars. ${\mathrm{CTDI}}_{w}$ values are calculated by the ${\mathrm{CTDI}}_{w}$ equation using thimble chamber measurements (19.61 mGy for adult, 20.93 mGy for adolescent, and 24.99 mGy for child) and pencil chamber measurements (17.70 mGy for adult, 19.86 mGy for adolescent, and 22.43 mGy for child) at L=100  mm, respectively. The error bars of all three $D_{\mathrm{eq}}$ values are overlapping for adolescent and child, respectively; and the ${\mathrm{CTDI}}_{w}$ values calculated using the thimble chamber measurements are within the error ranges. For adult, the $D_{\mathrm{eq},c}$ and the ${\mathrm{CTDI}}_{w}$ by thimble chamber are different from each other and the $D_{\mathrm{eq},p}$ and $D_{\mathrm{eq},w}$ values. The standard ${\mathrm{CTDI}}_{w}$ method using the pencil chamber results in lower dose indices as compared to the AAPM method for all phantoms.

**Fig. 5:**
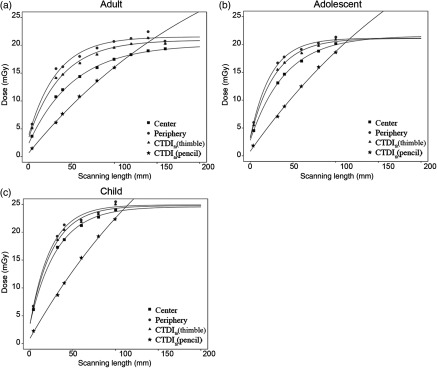
Toshiba MSCT scanner: comparison of AAPM curves fitted using the central and peripheral dose measurements by the thimble chamber with the AAPM curve fitted using the ${\mathrm{CTDI}}_{w}$ values calculated from dose measurements by the thimble chamber for the (a) adult, (b) adolescent, and (c) child head phantoms. The AAPM fits using the standard ${\mathrm{CTDI}}_{w}$ values measured by the pencil chamber are also shown. All dose measurements are normalized to 100 mAs. The values of the three parameters $D_{\mathrm{eq}}$, $L_{\mathrm{eq}}$, and α are summarized in Table 2(a).

As shown in Table 1, the scatter dose contribution percentages for adult, adolescent, and child are calculated by dividing Eq. (4) by Eq. (2). The same trend is observed in all three phantoms: the contribution of scatter radiation begins to demonstrate an asymptotic behavior as the scan length approaches 100 mm. The $D_{\mathrm{eq}}$ values from the fit [Eq. (2)] and the ${\mathrm{CTDI}}_{w}$ values are illustrated in Fig. 4. Measurements in the child head phantom had the highest radiation dose and the adult head phantom the least. For the child and adolescent phantoms, not only are the $D_{\mathrm{eq},c}$, $D_{\mathrm{eq},p}$, and $D_{\mathrm{eq},w}$ values similar to each other, but they are also similar to the ${\mathrm{CTDI}}_{w}$ value calculated from the dose measurement by the thimble chamber at 100-mm scan length. It is necessary to emphasize that, although the resulting values are similar for adults, the $D_{\mathrm{eq},c}$ is not within the error range of either the $D_{\mathrm{eq},p}$ or the $D_{\mathrm{eq},w}$, and the ${\mathrm{CTDI}}_{w}$ (thimble chamber) is lower than the $D_{\mathrm{eq},c}$. The ${\mathrm{CTDI}}_{w}$ obtained by the pencil chamber is about 10% lower than its corresponding $D_{\mathrm{eq}}$ for all phantoms. Figure 5 compares the AAPM fits using thimble chamber measurements and the fit using pencil chamber measurements for the Toshiba MSCT. In particular, the three thimble chamber plots are similar in shape, whereas the pencil chamber excludes scatter dose contribution and thus produces a relatively straight line.^2^ The numerical fitting results of the parameters are summarized in Table 2(a).

**Table 1:** Scatter dose percentages for Toshiba MSCT scanner (left) and i-CAT Next Generation dental CBCT scanner (right) in adult, adolescent, and child head phantoms at different scan lengths. The scatter dose percentages are calculated using the three parameters ($D_{\mathrm{eq}}$, α, and $L_{\mathrm{eq}}$) from the AAPM fit [Eqs. (2)–(4)] in the central hole.

Toshiba MSCT scanner				i-CAT Next Generation dental CBCT scanner			
Scan length (mm)	Adult scatter dose %	Adolescent scatter dose %	Child scatter dose %	Scan length (mm)	Adult scatter dose %	Adolescent scatter dose %	Child scatter dose %
4	34.91	38.52	40.28				
32	76.95	78.47	78.31				
40	79.60	80.73	80.28	40	75.66	74.12	62.67
60	83.29	83.77	82.82	60	80.04	78.45	67.73
80	85.14	85.20	83.92	80	82.28	80.64	70.30
100	86.18	85.95	84.44	100	83.58	81.88	71.75
120	86.81	86.38	84.71	110	84.02	82.30	72.23
140	87.21	86.63	84.85	130	84.67	82.89	72.89

**Table 2:** Summary of the three parameters $D_{\mathrm{eq}}$, $L_{\mathrm{eq}}$, and α from the AAPM fit in (a) Toshiba MSCT scanner and (b) i-CAT Next Generation dental CBCT scanner. (a) For each patient group, the $D_{\mathrm{eq}}$ values from thimble chamber measurements are very close to each other, with the highest being present in the child head phantom and the lowest in the adult head phantom. The pencil chamber measurements generate larger $D_{\mathrm{eq}}$, α, and $L_{\mathrm{eq}}$ values. (b) The AAPM method and the two Sedentex methods result in similar $D_{\mathrm{eq}}$, α, and $L_{\mathrm{eq}}$ values for each patient group.

(a) Toshiba MSCT scanner						
	Methods	Center	Periphery	CTDI Fit	CTDI Fit	
Phantoms		thimble chamber	thimble chamber	thimble chamber	pencil chamber	
Adult	$D_{\mathrm{eq}}$ (mGy)	20.13±0.19	21.53±0.48	20.93±0.40	45.08±8.13	
	α	0.88±0.0098	0.81±0.044	0.83±0.032	0.98±0.0033	
	$L_{\mathrm{eq}}$ (mm)	210.7±7.27	132.09±16.09	153.55±14.33	942.7±214.2	
Adolescent	$D_{\mathrm{eq}}$ (mGy)	21.55±0.40	21.14±0.43	21.08±0.45	54.87±11.36	
	α	0.87±0.013	0.84±0.030	0.85±0.025	0.98±0.0034	
	$L_{\mathrm{eq}}$ (mm)	162.77±9.47	97.11±9.66	114.99±10.28	995.3±256.6	
Child	$D_{\mathrm{eq}}$ (mGy)	24.58±0.40	24.92±0.85	24.77±0.72	57.95±12.15	
	α	0.85±0.016	0.86±0.050	0.85±0.037	0.98±0.0049	
	$L_{\mathrm{eq}}$ (mm)	126.27±7.93	97.93±16.14	106.63±13.77	840.5±227.8	
(b) i-CAT Next Generation						
	Methods	Center	Periphery	CTDI fit	Sedentex-DI1	Sedentex-DI2
Phantoms		thimble chamber	thimble chamber	thimble chamber	thimble chamber	thimble chamber
Adult	$D_{\mathrm{eq}}$(mGy)	12.51±0.34	12.15±0.22	12.27±0.25	12.53±0.17	12.33±0.27
	α	0.86±0.055	0.61±0.042	0.70±0.044	0.75±0.028	0.74±0.046
	$L_{\mathrm{eq}}$ (mm)	226.86±28.36	212.92±27.73	218.63±26.67	221.19±16.09	221.02±26.73
Adolescent	$D_{\mathrm{eq}}$ (mGy)	13.58±0.26	12.83±0.17	13.08±0.18	13.30±0.07	13.20±0.20
	α	0.84±0.054	0.59±0.031	0.68±0.035	0.70±0.012	0.72±0.039
	$L_{\mathrm{eq}}$ (mm)	202.94±21.27	210.97±20.67	207.46±19.43	220.14±7.27	206.11±19.60
Child	$D_{\mathrm{eq}}$ (mGy)	14.78±0.27	14.00±0.11	14.47±0.11	14.52±0.08	14.71±0.12
	α	0.74±0.072	0.51±0.021	0.57±0.019	0.61±0.016	0.60±0.019
	$L_{\mathrm{eq}}$(mm)	179.52±24.70	196.83±14.25	207.66±12.94	198.16±8.89	212.30±13.07

**Fig. 6:**
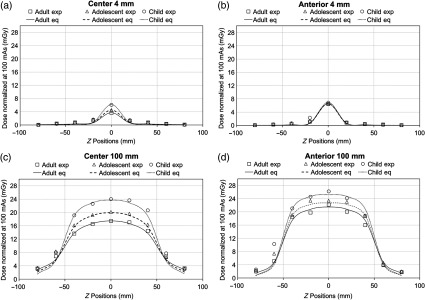
Toshiba MSCT center dose profiles with (a) L=4  mm and (b) L=100  mm and Toshiba MSCT anterior dose profiles with (c) L=4  mm and (d) L=100  mm for adult, adolescent, and child head phantoms. Dose estimation within z=±L/2 is fairly consistent. Greater difference between theoretical and experimental dose values is seen beyond z=±L/2. Dose profiles for 32-, 40-, 60-, and 80-mm scan lengths are not shown.

For dose measurements at z≠0, the experimental dose profiles match to the theoretical dose profiles calculated from Eq. (5). Figure 6 shows an example of the center and anterior dose profiles with L=4  mm and L=100  mm for all phantoms. Differences between theoretical and experimental values are mostly present around the ends of each scan length and beyond the scan length with dose variation of at least 1.2 mGy. Dose profiles plots for 32-, 40-, 60-, 80-mm scan lengths are not shown.

### 3.2 i-CAT Next Generation Dental Cone Beam Computed Tomography Scanner

**Fig. 7:**
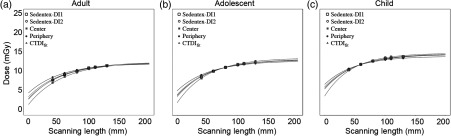
i-CAT Next Generation dental CBCT scanner: comparison of AAPM curves fitted using the central and peripheral dose measurements by the thimble chamber to the AAPM curves fitted using the ${\mathrm{CTDI}}_{w}$, Sedentex-DI1, and Sedentex-DI2 values calculated from dose measurements by the thimble chamber for the (a) adult, (b) adolescent, and (c) child head phantoms. All dose measurements are normalized to 100 mAs. The values of the three parameters $D_{\mathrm{eq}}$, $L_{\mathrm{eq}}$, and α are shown in Table 2(b).

**Fig. 8:**
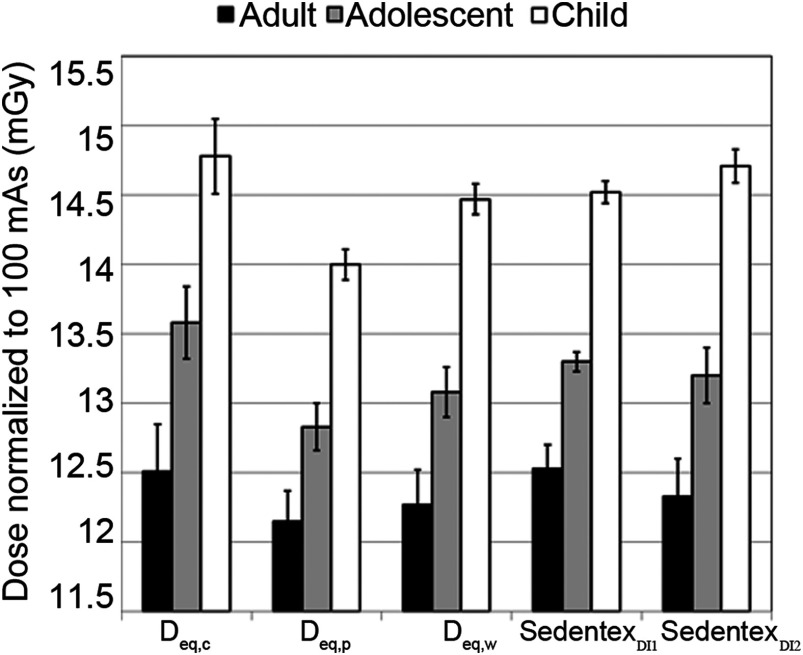
Comparison of $D_{\mathrm{eq}}^{\prime }s$ from the five AAPM fits in i-CAT Next Generation dental CBCT scanner. $D_{\mathrm{eq},c}$ and $D_{\mathrm{eq},p}$ are calculated from the AAPM equation [Eq. (2)] using the dose measurements taken in the central hole and the anterior hole by the thimble chamber, respectively. $D_{\mathrm{eq},w}$, Sedentex-DI1 $D_{\mathrm{eq}}$, and Sedentex-DI2 $D_{\mathrm{eq}}$ are calculated from the AAPM fit using the dose index values obtained from the ${\mathrm{CTDI}}_{w}$ equation and the two SEDENTEXCT DI methods.

The distribution of scatter radiation percentages for adult, adolescent, and child head phantoms from L=40 to 130 mm is shown in Table 1 (i-CAT Next Generation Dental CBCT Scanner). A similar trend is seen in which the behavior of scatter radiation percentage becomes asymptotic as the scan length reaches 100 mm in all three phantoms. The AAPM fit [Eq. (2)] curves are shown in Fig. 7 and the numerical fitting results of the parameters are shown in Table 2(b). Furthermore, the $D_{\mathrm{eq},c}$, $D_{\mathrm{eq},p}$, $D_{\mathrm{eq},w}$, Sedentex-DI1 $D_{\mathrm{eq}}$, and Sedentex-DI2 $D_{\mathrm{eq}}$ are compared in Fig. 8, which shows that the $D_{\mathrm{eq},c}$, $D_{\mathrm{eq},w}$, Sedentex-DI1 $D_{\mathrm{eq}}$, and Sedentex-DI2 $D_{\mathrm{eq}}$ are not different from each other for adult, adolescent, and child, respectively. However, for the child phantom, the error bars of the $D_{\mathrm{eq},p}$ values calculated are not overlapping with the other $D_{\mathrm{eq}}$ values. The ${\mathrm{CTDI}}_{w}$, Sedentex-DI1, and Sedentex-DI2 for three of the FOVs, summarized in Table 3, are very similar with a difference of 0.2 mGy at most.

**Table 3:** Summary of ${\mathrm{CTDI}}_{w}$, Sedentex-DI1, and Sedentex-DI2 values in Carestream 9300 and iCAT Next Generation dental CBCT scanners for adult, adolescent, and child. The ${\mathrm{CTDI}}_{w}$ calculations result in the highest dose indices for the three FOVs in CS 9300 dental CBCT scanner. The three dose indices are very similar in i-CAT NG Dental CBCT scanner, with a difference of 0.2 mGy at most. All doses are measured by the thimble ionization chamber. All the dose indices are normalized to 100 mAs and expressed in mGy.

Scan length (mm)	${\mathrm{CTDI}}_{w}$ normalized to 100 mAs (mGy)			Sedentex-DI1 normalized to 100 mAs (mGy)			Sedentex-DI2 normalized to 100 mAs (mGy)		
Carestream 9300									
	Adult	Adolescent	Child	Adult	Adolescent	Child	Adult	Adolescent	Child
60	11.12	9.00	10.98	11.03	8.65	7.78	10.81	7.78	10.87
110	12.92	12.16	11.84	12.24	11.70	10.70	12.67	10.70	11.82
135	12.58	10.35	11.45	12.33	10.31	9.06	12.29	9.06	11.37
i-CAT Next Generation									
	Adult	Adolescent	Child	Adult	Adolescent	Child	Adult	Adolescent	Child
60	9.39	10.22	11.84	9.33	10.15	11.88	9.23	10.16	11.84
110	11.14	12.05	13.47	11.23	12.04	13.53	11.11	12.11	13.59
130	11.42	12.41	13.78	11.61	12.42	13.88	11.40	12.39	13.94

**Fig. 9:**
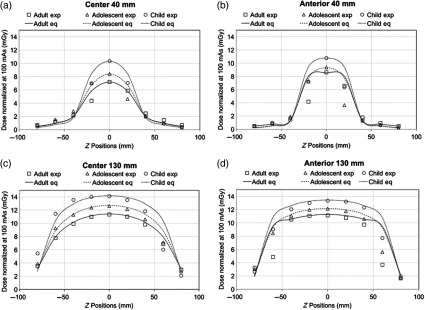
i-CAT Next Generation dental CBCT anterior dose profiles with (a) L=40  mm and (c) L=130  mm, and center dose profiles with (b) L=40  mm and (d) L=130  mm for the adult, adolescent, and child head phantoms. Similar to Fig. 6, inconsistency between theoretical and experimental dose values is primarily present at z=±L/2. Specifically, variations occur at z=±20  mm for L=40  mm and at z=±60 for L=130  mm. Theoretical dose estimations are fairly consistent with experimental dose measurements within and beyond z=±L/2. Dose profiles for 60-, 80-, 100-, and 110-mm scan lengths are not shown.

The dose profiles plotted using the three fitted parameters $D_{\mathrm{eq}}$, $L_{\mathrm{eq}}$, and α from Eq. (5) result in similar shapes to the profiles plotted by experimental dose measurements (Fig. 9). Variations between theoretical and experimental dose values predominantly exist around the edges of FOVs in which the experimental dose values are mainly lower than the theoretical predications by at least 1.0 mGy. Theoretical dose estimations are fairly consistent with experimental dose measurements within and beyond z=±L/2 with difference of at most 0.8 mGy. Dose profiles for 60-, 80-, 100-, and 110-mm scan lengths are not shown.

### 3.3 CS 9300 Dental Cone Beam Computed Tomography Scanner

**Fig. 10:**
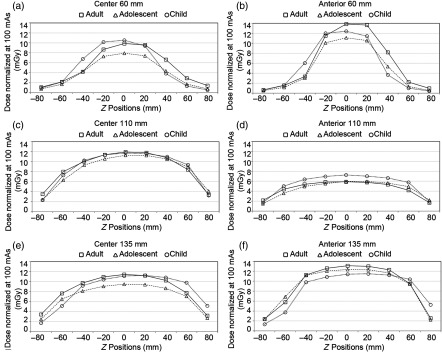
CS 9300 dental CBCT scanner center dose profiles along the z-axis with (a) L=60  mm, (c) L=110  mm, and (e) L=135  mm, and anterior dose profiles with (b) L=60  mm, (d) L=110  mm, and (f) L=135  mm cm for adult, adolescent, and child head phantoms.

The AAPM fit equation [Eq. (2)] requires at least four dose measurements at four different scan lengths to approximate values of the three parameters ($D_{\mathrm{eq}}$, $L_{\mathrm{eq}}$, and α); however, CS 9300 dental CBCT scanner only offers three FOVs covering the full diameter of the adult head, which is unsuitable for use with the AAPM method. Consequently, only the ${\mathrm{CTDI}}_{w}$, Sedentex-DI1, and Sedentex-DI2 values are computed for each FOV using dose measurements from the central hole of the phantom for all three phantoms (Table 3). The ${\mathrm{CTDI}}_{w}$ calculation results in the highest dose index values as compared to the two SEDENTEXCT DI methods. In addition, the measured dose profiles along the z-axis for all the FOVs are shown in Fig. 10.

## 4 Discussion

Several studies have addressed the limitations of the standard CTDI metric with advanced CT technologies^3^^–^^7^ including helical scanning and CBCT acquisition as compared to the AAPM methodology. Using adult and pediatric phantoms, we have not only further examined the advantages and disadvantages between the CTDI and the AAPM methodologies on the Toshiba MSCT scanner for different patient demographics but also implemented and compared the AAPM^4^ method with the CTDI method and the SEDENTEXCT DI method^14^ on two dental CBCT scanners. From the practical perspective, the AAPM method is extremely time-consuming and relatively difficult to perform in a clinical setting, though it produces accurate results. The time spent on performing the AAPM method is about 2 h on average for each phantom, whereas the CTDI method can be performed on all three phantoms within just 1 h. To overcome the difficulty posed by the AAPM method, we suggest an alternative that consumes much less time and approximates the AAPM result well, which is to utilize the ${\mathrm{CTDI}}_{w}$ equation to weight the center and peripheral dose measurements by the thimble chamber in a 100-mm scan length for pediatric phantoms or 160-mm scan length in adult phantoms to approximate the AAPM center $D_{\mathrm{eq}}$.

### 4.1 Multislice Computed Tomography Scanner

We have performed replicate measurements at the central and off-centered planes on the adult phantom according to Deman et al.^2^ to show that the resulting $D_{\mathrm{eq}}$ values from fitting with the AAPM equations [Eqs. (2)–(4) and (5)] are indeed reproducible. Specifically, we have included the thimble chamber dose measurements up to 160-mm scan length on the adult phantom, and the resulting $D_{\mathrm{eq},c}$ and $D_{\mathrm{eq},p}$ are 20.13±0.19  mGy and 21.53±0.48  mGy compared to 20.69±0.71  mGy and 22.61±0.52  mGy in Deman et al.’s work.^2^ Also, the scatter dose percentage on the z-axis at 100-mm scan length is slightly lower at 84.46% according to Deman et al.^2^ comparing to our measurement 86.18%, and similarly an asymptotic behavior has been observed with the scatter dose contribution in both studies as the scan length reaches 100 mm. Since an increased patient volume generates more scattered radiation,^15^ we hypothesized that the $D_{\mathrm{eq}}$ measured in the adult phantom would be acquired with a longer scan length compared to the child and the adolescent head phantoms, whose $D_{\mathrm{eq}}^{\prime }s$ would be reached with shorter scan lengths, as smaller volumes produce less scattered radiation. Therefore, we have only measured up to 100-mm scan length instead of 160 mm to improve experimental efficiency for the two pediatric phantoms. Indeed, the scatter radiation percentages in smaller volumes (child and adolescent) resulted in reduced scatter contributions (Table 1), and the AAPM fit curves for adolescent and child became asymptotic faster as compared to adult (Fig. 5).

Figure 4 visually compares the values of $D_{\mathrm{eq},c}$, $D_{\mathrm{eq},p}$, $D_{\mathrm{eq},w}$, and ${\mathrm{CTDI}}_{w}$ using the thimble chamber measurements and ${\mathrm{CTDI}}_{w}$ by pencil chamber at L=100  mm for adult, adolescent, and child, respectively. For adolescent and child, the $D_{\mathrm{eq},c}$, $D_{\mathrm{eq},p}$, and $D_{\mathrm{eq},w}$ values are not only similar to each other but also similar to the ${\mathrm{CTDI}}_{w}$ value by the thimble chamber, so we can faithfully conclude that the ${\mathrm{CTDI}}_{w}$ by thimble chamber from measurements over a 100-mm scan length is a close representation of the $D_{\mathrm{eq}}$.

However, the ${\mathrm{CTDI}}_{w}$ by the thimble chamber at L=100  mm for adult is lower than the $D_{\mathrm{eq},c}$, $D_{\mathrm{eq},p}$, and $D_{\mathrm{eq},w}$ values. Such behavior is expected because more scatter buildup would be produced as the phantom diameter increases.^16^ Therefore, 100-mm scan length is insufficiently long to include enough scatter radiation contribution for the adult head phantom and the calculated dose index cannot closely represent the AAPM $D_{\mathrm{eq}}$ values. A longer scan length such as L=160  mm results in a dose index value of 20.28 mGy, which is similar to the $D_{\mathrm{eq},c}$ obtained from the AAPM fit. Our measurements show that the ${\mathrm{CTDI}}_{W}$ using a thimble chamber will approximate the $D_{\mathrm{eq}}$ well for L=100  mm for pediatric phantoms and L=160  mm for adult phantoms.

### 4.2 i-CAT Next Generation Dental Cone Beam Computed Tomography Scanner

Various dosimetry studies have used thermoluminescent dosimeters with anthropomorphic RANDO head phantoms to examine the effective doses (mSv) in dental CBCT scanners.^17^^–^^20^ However, there have been limited studies published on absorbed dose for dental CBCT, not to mention absorbed dose studies on pediatric patients. Choi and Ford^13^ have validated the use of the two pediatric PMMA head phantoms to measure the absorbed doses with i-CAT NG CBCT and concluded the highest absorbed dose being observed in the smallest phantom and the lowest in the largest phantom, which is consistent with the trend of $D_{\mathrm{eq}}^{\prime }s$ from the AAPM fit shown in this study. For the i-CAT NG dental CBCT, the AAPM method is feasible to use for dose index determination in both adult and pediatric head phantoms.

There is no noticeable difference in the $D_{\mathrm{eq}}$ values between the five AAPM curves for adult or adolescent; the only difference is seen in the $D_{\mathrm{eq},p}$ for child (Fig. 8). The particular low $D_{\mathrm{eq},p}$ value for child could be owing to the small diameter of the child head phantom, which produces less scatter radiation.^16^ Although the $D_{\mathrm{eq},c}$, $D_{\mathrm{eq},w}$, Sedentex-DI1 $D_{\mathrm{eq}}$, and Sedentex-DI2 $D_{\mathrm{eq}}$ do not differ from each other, we recommend, with consideration of radiation safety, to use the highest dose index ($D_{\mathrm{eq},c}$) as the most conservative estimate of the true dose. To improve efficiency practically, the dose measured using the thimble chamber at the longest scan length (L=130  mm) can again represent $D_{\mathrm{eq},c}$ with an error of 9% for adult, 7% for adolescent, and 3% for child.

### 4.3 CS 9300 Dental Cone Beam Computed Tomography Scanner

The CS 9300 dental CBCT is a variable FOV scanner with a range of collimations from 13.5×17  cm for craniofacial imaging down to 5×5  cm for imaging individual teeth. Due to fixed collimation sizes and a limited number of FOVs provided by the scanner, the AAPM method is impracticable to perform in this case because the AAPM fit requires at least four dose measurements at different scan lengths with the same diameter size, but there are only three suitable FOVs for full diameter scan, which are 6×17  cm, 11×17  cm, and 13.5×17  cm, respectively. For the variable field-of-view dental CBCT scanners, where both the diameter and scan lengths change for different imaging tasks, the AAPM method is insufficient, identifying a flaw in what was meant to be a universal methodology for CBCT dosimetry. Many of the dental scanners currently available have a limited selection of preset scanning lengths, rendering the AAPM method impossible for dosimetry of many dental CBCT scanners. Therefore, we can only compare dose indices including ${\mathrm{CTDI}}_{w}$, Sedentex-DI1, and Sedentex-DI2 (Table 3), and plot the experimental dose profiles for the three FOVs at the central axis (Fig. 10). Although the three methods do not differ greatly from each other, we recommend to use the ${\mathrm{CTDI}}_{w}$ calculation with regard to radiation safety since it yields the highest dose indices.

Dose measurements were obtained under clinical settings, which include protocols using different kVp and mAs values for different-sized patients, which promote reduced doses for pediatric patients. Depending on the FOV and scan settings used, the axial dose distribution is changed correspondingly to ensure adequate coverage of the desired anatomical features, which complicates dose index determination.^21^ For example, Abouei et al.^11^ have described asymmetries between the right and the left for the 6×17-cm FOV used for imaging the temporal mandibular joint and between the posterior and the anterior for the 11×17-cm FOV used for craniofacial imaging, and a symmetric dose distribution for the 13.5×17-cm FOV, which is also used for craniofacial imaging. The dose indices calculated using our dose measurements by the thimble chamber show no difference between the three dose index methods for symmetric and asymmetric dose distributions. However, in the study by Araki et al.,^22^ a greater difference between SEDENTEXCT DI1 and DI2 is found for the 5×5-cm FOV (Sedentex-DI1: 1.825 mGy; Sedentex-DI2: 2.837 mGy), which could be attributed to (1) the absorbed doses are measured using the 10-cm pencil ionization chamber and (2) the small FOV size employs an off-centered FOV positioning to capture individual teeth, thereby altering the contributions from both the primary and scatter radiation.

## 5 Conclusion

This study not only investigates the CTDI and the AAPM methodologies on the Toshiba MSCT scanner but also implements and compares the AAPM method with the CTDI and the SEDENTEXCT DI methods on the i-CAT Next Generation dental CBCT scanner and CS 9300 dental CBCT scanner using adult, adolescent, and child head phantoms. The use of pediatric head phantoms confirms that the dose underestimation of the CTDI method is similar in different-sized patients compared with the AAPM method. An alternative dosimetry method for MSCT could be to utilize the ${\mathrm{CTDI}}_{w}$ equation with the thimble chamber measurements at the center and periphery for L=100  mm for pediatric phantoms and for L=160  mm for the adult phantom to calculate a “weighted $D_{\mathrm{eq}}$,” which could be a close representation of the AAPM center $D_{\mathrm{eq}}$. For i-CAT NG CBCT, the equilibrium dose at the central axis calculated by the AAPM method can be used for dose assessment. However, the AAPM method is not suitable for CS 9300 CBCT, because it does not offer enough concentric FOVs for the AAPM equation to calculate $D_{\mathrm{eq}}$. For dental scanners, we recommend using the dose index calculated by the ${\mathrm{CTDI}}_{w}$ equation instead of the SEDENTEXCT DI method for dose assessment, using measurements with a 2-cm thimble chamber. Although the AAPM method produces very accurate results as compared to the CTDI method, it is restricted in that (1) the fit requires at least four dose measurements to approximate $D_{\mathrm{eq}}$, α, and $L_{\mathrm{eq}}$; and (2) it is very time-consuming for medical physics to assemble the experimental setup and perform measurements.
